# Role of bile acids in inflammatory liver diseases

**DOI:** 10.1007/s00281-021-00869-6

**Published:** 2021-07-08

**Authors:** Ioannis Evangelakos, Joerg Heeren, Esther Verkade, Folkert Kuipers

**Affiliations:** 1 Department of Biochemistry and Molecular Cell Biology, University Medical Center Hamburg-Eppendorf, Martinistrasse 52, 20246 Hamburg, Germany; 2 Department of Pediatrics, University of Groningen, University Medical Center Groningen, Groningen, Netherlands; 3 Department of Laboratory Medicine, University of Groningen, University Medical Center Groningen, Groningen, Netherlands

**Keywords:** Bile acid signaling, Bile acids, Immune cells, Immunity, Inflammation, Liver, Liver disease, Microbiome, Non-alcoholic fatty liver disease, Primary biliary cholangitis, Primary sclerosing cholangitis

## Abstract

Bile acids and their signaling pathways are increasingly recognized as potential therapeutic targets for cholestatic and metabolic liver diseases. This review summarizes new insights in bile acid physiology, focusing on regulatory roles of bile acids in the control of immune regulation and on effects of pharmacological modulators of bile acid signaling pathways in human liver disease. Recent mouse studies have highlighted the importance of the interactions between bile acids and gut microbiome. Interfering with microbiome composition may be beneficial for cholestatic and metabolic liver diseases by modulating formation of secondary bile acids, as different bile acid species have different signaling functions. Bile acid receptors such as FXR, VDR, and TGR5 are expressed in a variety of cells involved in innate as well as adaptive immunity, and specific microbial bile acid metabolites positively modulate immune responses of the host. Identification of Cyp2c70 as the enzyme responsible for the generation of hydrophilic mouse/rat-specific muricholic acids has allowed the generation of murine models with a human-like bile acid composition. These novel mouse models will aid to accelerate translational research on the (patho)physiological roles of bile acids in human liver diseases .

## Introduction

Bile acids are cholesterol metabolites that are exclusively produced in the liver by a complex, multiple-step process, involving cytosolic, mitochondrial, and peroxisomal enzymes [1]. These amphipathic molecules are present in all vertebrate species, with variations on a general structural theme: C24 and C27 bile acids together with C27 bile alcohols are considered to constitute most of the bile acid family, as extensively reviewed by Hagey and Hofmann [2]. Similar molecules exist across the entire animal kingdom, as exemplified by a molecule called dafachronic acid that serves bile acid–like functions in the worm *C. elegans* [3]. This broad prevalence underscores the wide variety of biological functions in the body that are covered by bile acids and bile acid look-alikes. In addition to their “classical” roles in the generation of bile, intestinal absorption of dietary lipids and proteolytic cleavage of dietary proteins, antimicrobial activities in the small intestine, and cholesterol homeostasis, a series of important physiological bile acid functions have been discovered during the past two decades. It is now well-established that bile acids exert hormone-like functions in the control of glucose, lipid, and energy metabolism, in cellular proliferation, in the control of the detoxification reactions, as well as in the modulation of the immune system [4–7]. As summarized in Table 1, these actions are mediated through activation of the nuclear receptors farnesoid X receptor (FXR), pregnane X receptor (PXR), constitutive androstane receptor (CAR), vitamin D receptor (VDR), liver X receptors α/β (LXRα/β), and RAR-related orphan receptor γt (RORγt) [8, 9] as well as membrane-bound G protein-coupled receptors Takeda G protein-coupled receptor 5 (TGR5 aka G protein-coupled bile acid receptor 1 (GPBAR1)), sphingosine-1-phosphate receptor 2 (S1PR2), and muscarinic acetylcholine receptor M2 and M3 (CHRM2/3) (Table 1) [10]. Some bile acid–signaling pathways, particularly FXR and TGR5, have been recognized as bona-fide drug targets for the treatment of cholestatic (e.g., PBC, PSC) and metabolic (NAFLD/NASH) liver diseases [11]. In fact, the FXR agonist obeticholic acid (OCA) has been FDA-approved for specific cases of PSC in 2016 [12, 13] and a series of other molecules is in advanced clinical development.

**Table 1:**
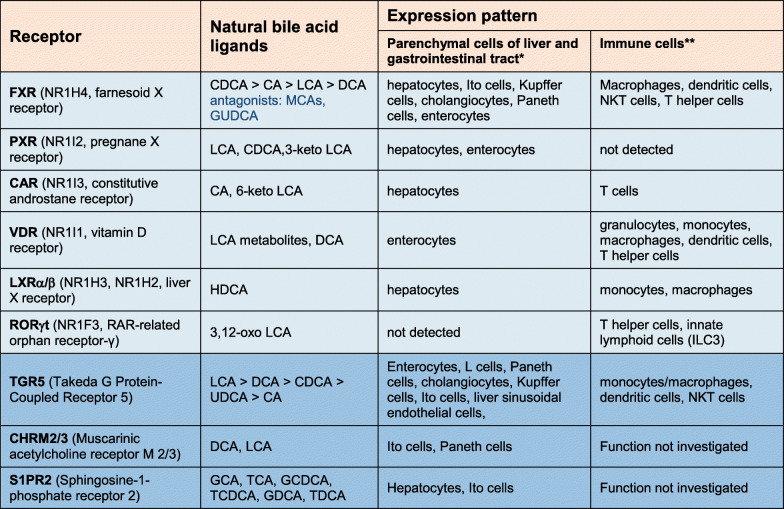
Expression pattern of nuclear and membrane-bound bile acid receptors (adapted according to [7]

*Cell type specificity is based on RNA single cell sequencing of human samples (https://www.proteinatlas.org/)

**Only immune cell types in which a functional role of the corresponding bile acid signaling pathway was demonstrated are listed

*CA* cholic acid*; UDCA* ursodeoxycholic acid*; BA(s)* bile acid(s)*; ALT* alanine aminotransferase*; AST* aspartate aminotransferase*; norUDCA* nor-ursodeoxycholic acid*; NEMO* NF-kappa-B essential modulator*; OCA* obeticholic acid*; Mc4r* Melanocortin 4 receptor*; WT* Western type*; HF* high fat*; AMLN* Amylin Liver NASH model*; DIO* diet-induced obesity*; MCD* methionine-choline deficient*; a-SMA* a-smooth muscle actin; *CK19* cytokeratin 19; STAM *streptozotocin-administered mice*; *TG* triglycerides; *CDHF* choline-deficient high fat; *LDL* low-density lipoprotein; *HDL* high-density lipoprotein; *GGT* gamma-glutamyltransferase; *T2DM* type 2 diabetes mellitus; *ACC* acetyl-CoA carboxylase; *CCR2/5* chemokine receptor type 2/5; *FGF19* fibroblast growth factor 19; *W* weeks; *M* months

**Fig. 1:**
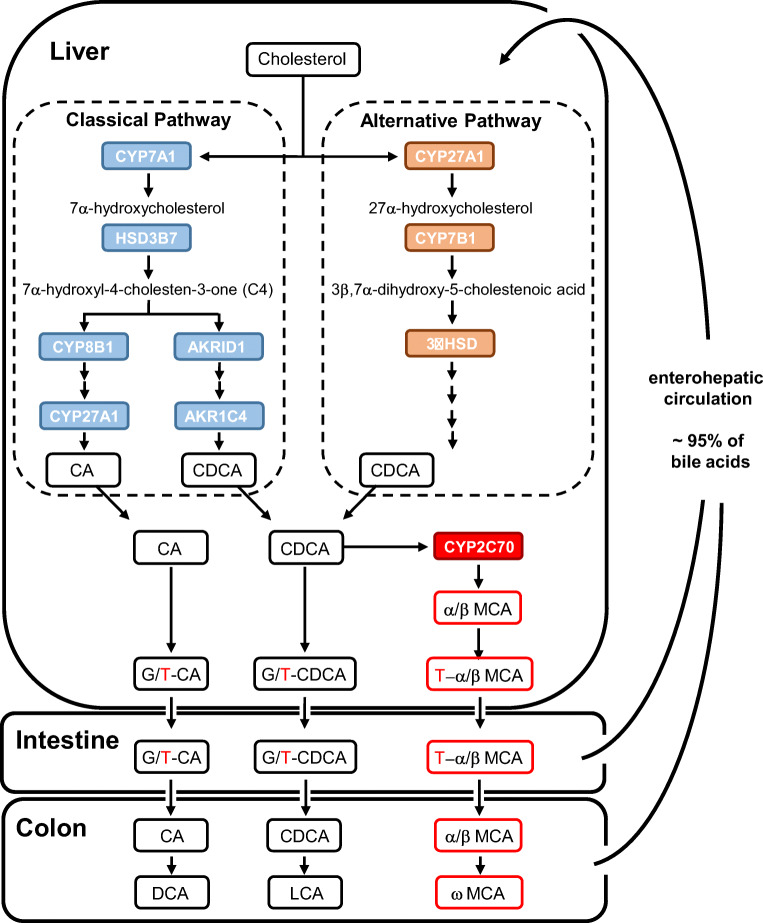
Generation of primary and secondary bile acids. Cholesterol is converted by a series of oxidative reactions to the primary bile acids, cholic acid (CA), and chenodeoxycholic acid (CDCA). In response to a meal, the conjugated forms of primary bile acids are released into the small intestine where they play an important role in digestion of dietary lipids. In the ileum of the intestine, approximately 95% of bile acids are reabsorbed and return to the liver via the enterohepatic circulation. In the colon, primary bile acids are deconjungated and converted by a number of bacterial enzymes to secondary bile acids such as deoxycholic acid (DCA) or lithocholic acid (LCA), which can be excreted or follow the enterohepatic circulation. As indicated in red, in mice bile acids are primarily conjugated to taurine (T), while human bile acids are conjugated to glycine (G). Notably, murine bile acids known as muricholic acids (MCA) are generated by CYP2C70, an enzyme expressed in murine but not in human liver explaining the difference in the composition of human and murine bile acid species

This review focuses on the role(s) of bile acids in the inflammatory components of cholestatic and metabolic liver diseases. Under certain conditions, bile acids may initiate immune reactions through their detergent actions leading to cellular damage or modulate immune reactions via bile acid receptors that are expressed in various cell types of the immune system. It is important to realize that the differences in bile acid structure that exist between species but also within species have a strong impact on their detergent actions as well as on their capacity to activate the various receptors mentioned. Hence, the concentration at the site of activation and the composition of the bile acids are important parameters to consider. For the purpose of this review, we will concentrate on bile acids that are present in humans and in mice/rats, as most pre-clinical studies on liver diseases have been conducted in these species. In humans, the primary bile acids cholic acid (CA) and chenodeoxycholic acid (CDCA) are synthesized in the liver and can be converted by intestinal bacteria into secondary deoxycholic (DCA) and lithocholic (LCA) acids and a number of less abundant metabolites, which can all be re-absorbed from the intestine (see Fig. 1 and the next section). Thus, the human bile acid pool consists of a mixture of primary and secondary bile acids with different physicochemical characteristics. It is therefore of importance to realize that the composition of the (healthy) human bile acid pool, as reflected in the systemic circulation, shows very large intra-individual variations [14, 15] and that this composition is modulated in various disease states, e.g., in type 2 diabetes [16]. In addition, it must be realized that that the composition of the murine bile acid pool fundamentally differs from that of humans. Activity of the Cyp2c70 enzyme, which is only present in mouse and rat livers, rapidly converts di-hydroxylated CDCA into tri-hydroxylated muricholic acids (MCAs), which account for ~ 35% of the total bile acid pool in mice [17]. As a consequence, the murine bile acid pool is much more hydrophilic than that of humans. Given the fact that the various bile acids have dissimilar affinities for the activation of the bile acid receptors (see Table 1) while MCAs as well as the human secondary ursodeoxycholic acids (UDCA) act as FXR and TGR5 antagonists [18], this species difference between mice and humans will modulate the activation state of bile acid receptors and, in turn, affect metabolism of nutrients, hormone secretion, and immune system. Therefore, translation of results from rodent studies to the human situation is challenging. Recently, a novel mouse model with human-like bile acid pool has been developed by depleting the MCA-generating enzyme *Cyp2c70* [19]. These *Cyp2c70-*deficient mice presumably serve as a better model for research on the role of bile acids in liver diseases [20–22].

During the past years, bile acid–mediated activation of TGR5, FXR, and VDR has been implicated in shaping of innate immune responses [23]. Indeed, the membrane-bound receptor TGR5 was first identified and characterized in human and rabbit macrophages and monocytes [24]. A series of very recent papers has now also identified specific roles of bile acids and bile acid receptors in the adaptive immune system [25–27], particularly by directly modulating the balance of Th17 and T_reg_ cells in the intestinal lamina propria by specific secondary bile acids in relation to inflammatory bowel diseases. We will attempt to integrate this knowledge while discussing the role of bile acids and bile acid receptors in inflammatory liver diseases.

## Synthesis, microbial conversion, and enterohepatic circulation of bile acids

Primary bile acids, i.e., CA and CDCA in humans and CA, αMCA, βMCA, and UDCA in mice, are synthesized in the liver in a series of enzymatic modifications of the cholesterol molecule via the so-called classical/neutral or the alternative/acidic pathway, respectively (Fig. 1). Both pathways are under stringent and complex modes of control, delineating the (patho)physiological importance of these synthetic cascades [see [28, 29] for review]. To further increase solubility, newly synthesized bile acids are conjugated with hydrophilic molecules such as glycine or taurine. In humans, the majority of bile acids are glycine-conjugated and a small fraction is tauro-conjugated [30]. Bile acids of mice are almost exclusively tauro-conjugated, with a small amount of glycine conjugation. Conjugated bile acids are then actively secreted by the hepatocytes into bile canaliculi, transported via the bile ducts and stored in the gallbladder. Upon food ingestion, cholecystokinin triggers contraction of the gallbladder and disposal of bile into the duodenum. In the small intestine, bile acids facilitate the absorption of lipid-soluble molecules but also act as signaling molecules, for example, by promoting TGR5-mediated secretion of GLP-1 by endocrine L-cells. In the ileum, the majority of bile acids is actively taken up by enterocytes and expelled into the portal circulation. Upon their return to the liver, bile acids are efficiently taken up by hepatocytes by specialized transporter proteins and, with or without further modifications, re-secreted into the bile to complete their enterohepatic circulation. A small part of bile acids escapes first-pass clearance by the liver and enters the peripheral circulation, particularly in the postprandial phase, and is then able to interact with bile acid receptors that are present in peripheral organs and tissues, such as the heart, adrenals, and adipose tissues [7, 31]. The transporter proteins involved in enterohepatic cycling of bile acids and their modes of regulation have largely been identified in the period 1990–2010 [see [4, 17] for reviews].

A certain fraction of bile acids per cycle is not taken up by the ileum and enters the colon where their structure can be modified by the gut microbiome through initial deconjugation and subsequent oxidation, dehydroxylation, and epimerization reactions which produce the so-called secondary bile acids. The most abundant secondary bile acid species are DCA and LCA in humans and mice while ω-muricholic acid (ωMCA) is only produced in mice. In addition, a wealth of other (intermediary) bile acid species can be found in colonic contents and in feces, such as isoallo-LCA, 3-oxo-LCA, or 7-oxo-DCA. These species were found to particularly contribute to intestinal immunity [25–27]. The unconjugated secondary bile acids can be absorbed from the colon, likely by passive means, to be transported to the liver and taken up by hepatocytes for re-secretion in the bile. As a consequence, the circulating bile acid pool consists of a mixture of primary and secondary bile acids. With each cycle, about 5% of the bile acid pool is lost in the feces and compensated for by de novo synthesis in the liver to maintain the bile acid pool. The human bile acid pool (in the order of 1–2 g) cycles 8–10 times per day, and 400–600 mg bile acids is newly synthesized each day to compensate for fecal bile acid loss, which strongly contributes to whole-body cholesterol turnover. Importantly, bile acid synthesis rates and pool sizes show large inter-individual variations in humans [32, 33] and, partly as a consequence hereof, there is a strong variability in plasma bile acid concentration and composition between individuals [14, 15]. In view of the “hormonal actions” of bile acids, this variability has to be taken into account in therapeutic strategies that interfere with bile acid signaling pathways.

## Interactions between bile acids and immune cells

Bile acid receptors come in two flavors, i.e., the nuclear and the cell surface receptors, with different modes of action, ligand specificities, and distribution patterns within the body. It is evident that activation of the nuclear receptors, i.e., FXR, and the more promiscuous receptors VDR, CAR, and PXR, requires entry of bile acids into the cell which, particularly for hydrophilic bile acid species, involves the activity of specific transporter proteins. Upon their activation, these nuclear receptors modulate expression of a series of target genes in an organ and cell type–specific context [see [4, 5] for review]. Cell surface receptors such as TGR5 can be activated without entering the cell to trigger activation of an adenylate cyclase and thereby increase intracellular cAMP and protein kinase A (PKA), which subsequently will induce a series of cell type–specific reactions. Bile acid receptors are expressed by a number of parenchymal cells and also immune cells, indicative for a role of bile acids in immune modulation. Initially, expression of FXR and TGR5 as well as of VDR and LXRs has been described in cells of the innate immune system, i.e., monocytes and macrophages, dendritic cells and natural killer (NK) cells, as well as NKT cells [7]. T cells were generally regarded as negative for FXR and TGR5, but recent studies have clearly demonstrated important roles for bile acid signaling via VDR as well as FXR also in adaptive immunity [26, 27], so far particularly in relation to inflammatory bowel disease. Activation of FXR and of TGR5 in macrophages, dendritic cells, and NKT has been shown to induce effects that are in general inhibitory in nature, and both receptors appear to be involved in the maintenance of tolerance of the hepatic immune system towards antigens and xenobiotics originating from the intestine. Both FXR^-/-^ and TGR5^-/-^ mice develop low-grade inflammation with age and are prone to develop inflammation when treated with infectious agents, illustrating the role of these receptors in providing signals that counteract macrophage effector functions. While both FXR and TGR5 are expressed in circulating monocytes and monocyte-derived macrophages, it appears that TGR5 is the dominant receptor in resident macrophages of the liver, i.e., Kupffer cells. Detailed descriptions of the modes of action by which FXR and TGR5 modulate innate immune reactions are beyond the scope of the overview and have recently extensively been reviewed [7]. In short, FXR can act through trans-repression of inflammatory genes by both SHP (short heterodimer partner)-dependent and SHP-independent means. SHP, whose expression is strongly induced by FXR, acts as a co-repressor at the promoter of FXR target genes and has also been shown to prevent binding of AP1 and p65 to promoters of inflammatory genes and to inhibit lncRNA H19, which induces expression of pro-inflammatory mediators Il-4 and Il-27 in models of cholestasis. In a SHP-independent manner, ligand-activated FXR is recruited to iNOS and Il-1β promoters to stabilize NCoR1 complexes and hence *trans*-represses expression of these genes. Likewise, TGR5 activation negatively regulates the expression of inflammatory signals. It has been demonstrated that TGR5 ligands, such as the secondary bile acids DCA and LCA, activate PKA and subsequent recruitment of CREB to the promoters of target genes which, for instance, reduces the activity of NF-κB. Both in the liver and intestine, TGR5 activation shifts macrophages from a M1 pro-inflammatory phenotype to a M2 anti-inflammatory phenotype. It has been demonstrated that treatment of *db/db* mice, a genetic model for obesity with MAFLD, with a dual FXR/TGR5 agonist improves liver histology and increases M2 macrophage markers in liver while a selective TGR5 agonist had similar beneficial effects in high fat diet induced MAFLD in mice. Hence, it appears that (specific) bile acids may act as endogenous modulators of macrophage polarization. The relevance of this process in humans awaits further studies.

Recently, an important link between microbial bile acid metabolism and adaptive immunity has also been described [34]. Hang et al. [26] identified two metabolites of LCA, 3-oxo-LCA and isoallo-LCA, as T cell regulators. Interestingly, 3-oxo-LCA suppressed differentiation of T helper (Th17) cells by directly binding to the transcription factor RORγt, while isoallo-LCA promoted differentiation of regulatory T (T_reg_) cells by stimulation of mitochondrial ROS production leading to increased expression of forkhead box P3 (FOXP3), the master transcriptional regulator of T_reg_ differentiation. Importantly, feeding of mice with either 3-oxo-LCA or isoallo-LCA reduced Th17 and increased T_reg_ differentiation, respectively, in the ileal lamina propria. Song et al. [25] reported that diet-dependent induction of a distinct population of FOXP3^+^ Treg cells that express RORγ, i.e., RORγ^+^- T_reg_ cells, that are critical in the maintenance of immune homeostasis in the colon, is mediated by secondary bile acids via activation of VDR. Indeed, VDR is highly expressed in the RORγ^+^T_reg_ population and LCA and 3-oxo-LCA are potent activators of VDR. The pathophysiological relevance of this bile acid-VDR signaling axis was demonstrated by showing that mice lacking VDR were more vulnerable to dextran sulfate sodium (DSS)–induced colitis. More recently, Campbell et al. [27] reported that particularly 3β-hydroxydeoxycholic acid (isoDCA), a relatively low abundant secondary bile acid, has a strong ability to enhance differentiation of peripherally induced T_reg_ cells. isoDCA increased FOXP3 induction through its action on dendritic cells to diminish their immune-stimulatory properties, with involvement of isoDCA-FXR signaling in this process. It was shown that isoDCA-producing microbial consortia increased the numbers of RORγ^+^T_reg_ cells in the colon. In this context, it is also interesting to note that bile acid–dependent activation of constitutive androstane receptor (CAR) leads to the expression of xenobiotic transporters and detoxifying enzymes and thus protects CD4-positive T effector cells in the lamina propria against harmful effects of hydrophobic bile acids [35]. Interestingly, CAR activated by bile acids also promotes the expression of anti-inflammatory IL10 [35].

Overall, these studies underscore the (patho)physiological importance of microbiome-derived bile acids as signaling molecules, now also in the control of adaptive immunity. Whether this contributes to intestine-liver crosstalk in the control of liver functioning and/or disease development remains to be established. In this context, it is of note that the local activation of effector T cells resulted in a decreased production of hepatocytes of potentially harmful bile acids in the liver [36], underlining the mutual interaction between immune response and metabolism.

## Bile acids in cholestatic liver diseases

Cholestasis is operationally defined by a disturbance in bile flow either caused by mechanical obstruction of bile ducts or by hepatic transporter defects. During cholestasis, intrahepatic and plasma bile acid levels will increase and only limited amounts of bile acids will reach the intestine to be modified by the gut microbiome. This, in combination with disturbed hepatic bile acid synthesis [37], results in an altered bile acid composition and localization and hence to disturbed bile acid signaling during cholestasis. Evidently, bile acids influence the immune system during cholestasis and thereby affect disease progression through various complex pathways [38, 39]. This paragraph will elaborate on bile acid actions on immunity during cholestasis and on potential treatment options that target bile acid metabolism.

Disturbances in both immunity and bile acid metabolism are evident in primary biliary cholangitis (PBC) and primary sclerosing cholangitis (PSC). In both diseases, a strong inflammatory reaction in bile ducts causes progressive biliary fibrosis, leading to ascending cholestasis. PBC is characterized by a female predominance, the presence of anti-mitochondrial antibodies (AMAs), and loss of intrahepatic bile ducts. PSC, on the other hand, affects both the intrahepatic and extrahepatic bile ducts and is associated with inflammatory bowel disease in the majority of patients. In both PBC and PSC, recruitment of cells of innate and adaptive immunity can be detected in portal fields such as Th17 cells and monocytes [40]. Intestinal mucosal T cells have been hypothesized to be recruited to the liver in response to expression of gut-homing molecules and chemokines in hepatic sinusoids in PSC, which might explain its link with IBD [41]. In PBC, Th17 cell abundance increases during disease progression, leading to the release of IL17 which triggers chemotaxis and granulopoiesis [42]. In advanced fibrosis, Th17 cells will further accumulate in the liver. T_reg_ cells are decreased in PBC whereas follicular helper T cells (Tfh), which drive humoral immunity, are increased. Furthermore, innate immunity is induced with an activation of Kupffer cells, macrophages and an increase in natural killer T cells (NKT cells) in PBC, and an increased abundance of Kupffer cells and perisinusoidal macrophages in PSC [43]. Altogether, activation of immune cells in PBC and PSC leads to an immune balance skewed towards an inflammatory phenotype. The accumulation of cytotoxic, particularly hydrophobic bile acids in the liver of PBC and PSC patients further aggravates the immune response, contributing to the development of fibrosis and, possibly, progression to malignancy.

Sterile inflammation is also evident in other cholestatic conditions [38]. Increasing evidence supports a role of bile acids herein through multiple potential pathways. It is widely accepted that high bile acid levels inside the liver contribute to hepatocyte death [44]. Different pathways of cell death have been proposed to induce inflammation in the setting of cholestasis. For example, Afonso et al. showed that PBC patients and bile duct–ligated (BDL) mice, i.e., an established model of obstructive cholestasis, show increased “necroptosis” via expression of receptor-interacting protein 3 (RIP3) and phosphorylation of mixed lineage kinase domain-like protein (MLKL) in the liver. Indeed, RIP3-KO mice showed reduced inflammatory cell infiltration at 3 days and 2 weeks after BDL, although no effect on fibrosis development was seen [45].

High bile acid levels observed under cholestatic conditions have also been shown to directly damage hepatocytes, which results in the release of the High-Mobility Group Box 1 (HMGB1) protein. This damage-associated molecular pattern (DAMP) molecule can induce the secretion of pro-inflammatory cytokines such as TNFα and IL6 by binding to the toll-like receptor 4 further promoting a sterile inflammation [46]. Furthermore, bile acids have been associated with an increased expression of the major histocompatibility complex (MHC) class I proteins on both hepatocytes and cholangiocytes [47], which may lead to increased antigen presentation and activation of CD8+ cytotoxic T cells. Bile acids also influence inflammatory reactions independently of cell necrosis or apoptosis. For example, the administration taurine-conjugated CA to mouse hepatocytes in vitro has been shown to increase mRNA expression of various cytokines and adhesion molecules independent of cell death. Among these were MCP1 (Ccl2), MIP2 (Cxcl2) and ICAM1, which are known to attract neutrophils [48]. In BDL mice, the elevated expression of ICAM1 led to the infiltration of neutrophils into the liver parenchyma [49]. The neutrophils that extravasated from the sinusoids induced hepatocyte death through the secretion of reactive oxygen species (ROS), and, indeed, the number of extravasated neutrophils was decreased by 90% in ICAM1-deficient mice subjected to BDL with a concomitant decrease in ROS [49]. Of note, PBC patients also have increased levels of the soluble adhesion molecule ICAM1 in plasma [50]. Podevin et al. demonstrated that BDL and bile acids in vitro can reduce the biological activity of interferons and render NK cells defective [51]. Kupffer cells, i.e., the specialized liver-resident macrophages, are important regulators of immunity as they are first in line to respond to gut-derived pathogens and DAMPs. Upon activation, these cells will secrete pro- or anti-inflammatory cytokines and chemokines depending on their M1 or M2 activation state, respectively. Moreover, Kupffer cells can promote the transformation of hepatic stellate cells into myofibroblasts and they can induce inflammatory responses in other liver cell types [52]. The role of Kupffer cells in cholestasis is, however, still debatable. Depletion of Kupffer cells in models of cholestasis worsens liver damage in some studies, while attenuating it in others [52, 53]. Since the effects of bile acids on immune cells during cholestasis are increasingly recognized, therapeutics targeting bile acid metabolism may beneficially modulate immune functions. In 1994 ursodeoxycholic acid (UDCA), a very hydrophilic bile acid with FXR antagonizing actions, was approved as therapy for PBC and is still considered the first-in-line treatment [42]. Although UDCA significantly increases transplantation-free survival rate in PBC patients, approximately 40% of PBC patients do not respond adequately to UDCA treatment [54, 55]. In PSC patients, the therapeutic effects of normal to low concentrations of UDCA are still uncertain and may be limited to a decrease in plasma markers of liver dysfunction [56]. High dosages of UDCA are even related to an increased risk of adverse outcomes such as esophageal/gastric varices or liver transplantation [57].

Several mechanisms of action have been proposed underlying the therapeutic effects of UDCA. UDCA can inhibit the intestinal absorption of endogenous, hydrophobic bile acids and thereby increase the hydrophilicity of the bile acid pool [58]. UDCA induces choleresis by stimulation of cholangiocytic bicarbonate secretion, restoring the “bicarbonate umbrella” that protects cholangiocytes against bile acid–induced damage [59]. The “cholehepatic shunt theory” explains hypercholeresis induced by UDCA by an intrahepatic circulation route in which UDCA is reabsorbed from the bile by the biliary epithelium and returned to hepatocytes to be re-secreted into the bile, inducing bile acid–dependent bile flow with each round [60]. Existence of this cholehepatic shunt has not yet been demonstrated in patients. Furthermore, UDCA increases the expression of hepatobiliary transporters, possibly by alleviating ER stress [61], and is hypothesized to protect membranes of liver cells including hepatocytes.

UDCA has also multiple immune-modulating effects. For instance, UDCA decreases the expression of MHC class I and II proteins on bile duct epithelial cells in PBC patients and may thereby influence adaptive immunity [62]. UDCA also decreased nuclear DNA fragmentation, i.e. a sign of apoptosis, in cholangiocytes of PBC patients These anti-apoptotic actions of UDCA have been attributed to its modulatory effects on ER stress, protection of mitochondrial function and regulation of survival signaling pathways such as NF-κB, AKT, MAPK, and PI3K [63]. Furthermore, UDCA can restore and increase NKT cell activity in PBC patients via the reduction of prostaglandin E2 production [64]. PBC patients intolerant of treatment with UDCA or those with high-risk disease as evidenced by UDCA treatment failure should be considered for second-line therapy, of which OCA is the only currently licensed agent that is recommended by the Institute for Health and Care Excellence [53]. In fact, this implies addition of an FXR agonists to a FXR antagonistic treatment regimen. Part of the beneficial effect may actually be exerted at the level of the intestine, i.e., by restoring intestinal barrier function that is commonly disrupted during cholestasis. Indeed, bile duct–ligated rats exhibited decreased FXR pathway expression in both jejunum and ileum, in association with increased gut permeability and local and systemic recruitment of NK cells resulting in increased interferon-γ expression and bacterial translocation. Treatment with the FXR agonist INT-747 markedly decreased NK cells and interferon-γ expression, normalized permeability selectively in ileum which is associated with a significant reduction in bacterial translocation [65]. In experimental cholestasis, there appears to be a protective role for FXR in the gut-liver axis, whether this applies to human PBC remains to be shown. In fact, UDCA-resistant PBC patients treated with OCA showed improved liver function but also a dose-dependent increase in complaints of pruritus, which limits the use of this drug [66, 67].

## Bile acids in metabolic control—relation to MAFLD

Metabolic-associated fatty liver disease or MAFLD (a revised terminology for the commonly used term “non-alcoholic fatty liver disease” or NAFLD) [68] is a rapidly emerging liver disease that already affects almost one-fourth of the global population. MAFLD ranges from simple steatosis (hepatic fat accumulation) to aggravated steatohepatitis (NASH), characterized by inflammation, ballooning, and tissue damage/fibrosis [69]. If several control mechanisms are surpassed, the latter may progress to cirrhosis or even hepatocellular carcinoma (HCC) [70]. In contrast to the immune-associated liver disorders, the main cause of MAFLD development is a prolonged imbalance of glucose, lipid, and cholesterol metabolism [71]. Recent studies have shown that many of the metabolic processes involved can be regulated by bile acids. For example, hepatic FXR activation mitigates cholesterol conversion to bile acids via CYP7A1 inhibition, attenuates lipogenesis by modulating transcriptional activity of both sterol regulatory element-binding protein 1c and carbohydrate-responsive element-binding protein (ChREBP), and, at the same time, controls gluconeogenesis and stimulates glycogen synthesis [72]. Interestingly, bile acids exert opposite effects regarding insulin signaling in the postprandial phase, as they can stimulate or inhibit GLP1 production in the intestinal L cells, via TGR5 or FXR activation, respectively [73]. The bile acid receptors have been also associated with anti-inflammatory responses, while numerous studies highlight important alterations in the bile acid pool size and their enterohepatic circulation during inflammatory diseases such as steatohepatitis [74]. Therefore, the implication of bile acids in MAFLD development and progression is increasingly acknowledged, while differential activation or modulation of their receptors is under investigation for potential treatments and therapeutic interventions [11].

## MAFLD-associated alterations in bile acid metabolism in mice and humans

A number of studies have reported distinct differences in bile acid levels and composition in various biological samples of murine models as well as patients with MAFLD or steatohepatitis. For instance, taurine-conjugated βMCA) together with taurocholate (TCA) were specifically increased in the serum of a methionine-choline-deficient diet (MCD)–fed mice, an established model to induce fatty liver in rodents, while after methionine or choline supplementation, their levels were normalized 10 [75]. Recently, Suga et al. reported that total circulating bile acids are elevated in mice with diet-induced NASH and are associated with the degree of fibrosis [76]. Next to observations in animal models, human studies uncover important changes in bile acid homeostasis in relation to MAFLD. Of note, postprandial as well as fasting bile acid levels have been found to be significantly elevated in the serum of adult patients with NASH and relate with its severity [77–79]. These studies argue towards a systemic exposure to potentially cytotoxic bile acid species, which could eventually trigger liver injury and/or mediate the pathogenesis of the disease. In an effort to identify differences on bile acid species level, a metabolomic analysis of a NASH cohort revealed increased levels of serum TCA, GCA, and GDCA in comparison to healthy individuals [80]. Circulating TCA and GCA, along with both CDCA conjugates, were also elevated in biopsy-proven MAFLD as well as NASH patients [81]. In line with the previous observations, Nimer et al. recently described that in a cohort of NAFLD patients, plasma TCA and GCA are positively associated with increasing grades of inflammation and fibrosis, respectively [82]. Interestingly, despite the fact that bile acid measurements in liver tissue revealed characteristic changes during MAFLD to NASH progression, reported outcomes were quite contradictory [83, 84]. The rationale between these disparities could be attributed to technical limitations of the studies, since bile acids were extracted from whole hepatic tissues. Instead, a compartmentalized analysis of blood, biliary, or intracellular concentrations is needed in order to identify bile acid alterations in the liver that might be correlated with MAFLD/NASH. Next to primary bile acid species–related changes, specific alterations between primary and secondary bile acids have been identified in NASH patients, suggesting dynamic changes in the gut microbiota during the establishment of the disease. The ratio of primary to secondary bile acids is higher in NASH patients, while the ratio of conjugated to unconjugated seem to be relatively unaffected [81, 85]. However, Legry et al. underlined an association of increased primary bile acids with insulin resistance, but not with hepatic necroinflammation in a biopsy-proven NASH cohort of obese individuals [86]. Another study in MAFLD patients revealed specific changes of the gut microbiome and an increase of fecal bile acid concentrations that were related to the degree of hepatic fibrosis in non-obese individuals, but not in obese subjects [87]. The authors argue that finding no relation between fibrosis and bile acid alterations in the obese state might be explained by a masking effect that obesity already manifests as a significant determinant of commensal microbiota and bile acid synthesis.

The foundation of bile acid alterations during MAFLD development has extensively been studied in humans and mice, primarily focused on hepatic expression of bile acid synthesis–related genes as well as their targets within the enterohepatic circulation [17, 88]. The expression of CYP7B1 has been found elevated in NASH patients, while CYP8B1 was down-regulated, suggesting a possible shift towards the alternative pathway of bile acid synthesis during disease progression [84]. In contrast, in a murine model of HFD-induced NASH, Cyp7b1 was down-regulated [89] and when *Cyp7b1*^*-/-*^ mice were given the same diet, no significant differences were observed in relation to the NASH score, rendering the alternative pathway still ambiguously involved in MAFLD progression. Interestingly, environmental factors such as cold exposure leads to the specific induction of *Cyp7b1* under conditions of cholesterol-enriched diets, which results in increased plasma bile acid levels, as well as fecal excretion [90]. These systemic adaptations to increased energy expenditure are accompanied by distinct changes in gut microbiota, decreased hepatic lipid accumulation, and increased heat production, suggesting an important role of bile acids generated by the alternative synthesis route for MAFLD.

Many studies, on the other hand, have reported that CYP7A1 expression is increased in MAFLD patients, suggesting that the classical pathway of bile acid synthesis can be also activated [81, 86, 91]. Recently, Govaere et *a*l. conducted a transcriptomic analysis of liver biopsies in patients with MAFLD that were categorized according to disease severity and based on histopathogical evaluation [92]. It could indeed be validated that CYP7A1 is up-regulated in all MAFLD stages, with its expression reaching a peak early upon the onset of the disease but progressively decreasing as steatohepatitis advanced. These findings imply that changes in bile acid synthesis occur very early, so that treatments designed to target bile acid synthesis could be considered even at primary stages of the disease. However, it is still not clear whether the up-regulation of CYP7A1 and its respective products mediate the development or act protectively against some features of the disease. For example, *Cyp7a1*^*-/-*^ mice presented greater hepatic inflammation, fibrosis, and lipid accumulation upon an MCD diet than wild-type controls, while AAV-mediated overexpression of CYP7A1 reversed these detrimental effects [93]. Therefore, the authors support that CYP7A1-mediated cholesterol conversion to bile acids can reduce intrahepatic cholesterol accumulation and/or produce ligands for FXR or TGR5 with potent anti-inflammatory activity. The hepatoprotective activity of FXR has been supported by several animal studies performed in mice lacking FXR and TGR5, which present augmented MAFLD and steatohepatitis-related features [94, 95]. Interestingly, a study comparing liver versus intestine-specific *Fxr*^*-/-*^ mice underlined that the protective effect against lipid accumulation was mainly attributed to the hepatic presence of FXR and was rather independent of intestinal FGF15 activation [96]. In parallel, since *Tgr5*^*-/-*^ mice are prone to LPS-induced inflammation [97], it is postulated that TGR5 could exert significant anti-inflammatory properties during MAFLD and steatohepatitis, but this remains to be elucidated.

## Pre-clinical studies and human clinical trials interfering with bile acid signaling pathways for treatment of MAFLD

Molecular pathways of bile acid synthesis and signaling constitute attractive targets for therapeutic interventions in metabolic liver disorders [98, 99]. Therefore, bile acid supplementations and treatment with agonists or analogs for specific targets within their enterohepatic circulation are in the center of pre-clinical studies as well as ongoing clinical trials. A summary of relevant, up-to-date studies that have been conducted in relation to MAFLD and steatohepatitis in rodents and humans can be seen in the Tables 2 and 3, respectively. The selected studies for rodents refer to bile acids, FXR and TGR5 agonists, both selective and dual. For humans, FGF19 analog trials as well as combinatorial trials are additionally included. The steroidal FXR agonist obeticholic acid (OCA) has been advanced in phase III clinical trials as the most potent drug for MAFLD/steatohepatitis due to the positive outcomes for hepatic fibrosis and inflammation resolution [100–102]. Despite the desired outcomes, a common adverse effect of all these trials was a worsening of plasma lipid profile, attributed to increased LDL and low HDL cholesterol, which renders OCA treatment potentially atherogenic. For this reason, new generations of both steroid and non-steroid FXR agonists as well as combinations with lipid lowering compounds have been used in animal studies and advanced to clinical trials (Tables 2 and 3). Additionally, since FGF19 is a gut hormone induced by intestinal FXR and postulated to mediate some of its beneficial effects, the FGF19 analogue NGM282 has been advanced into clinical trials with promising outcomes so far (Table 3). Lastly, selective TGR5 and dual FXR/TGR5 agonists have been successfully used in pre-clinical animal studies, but so far none of them had been advanced to clinical trials.

**Table 2:**
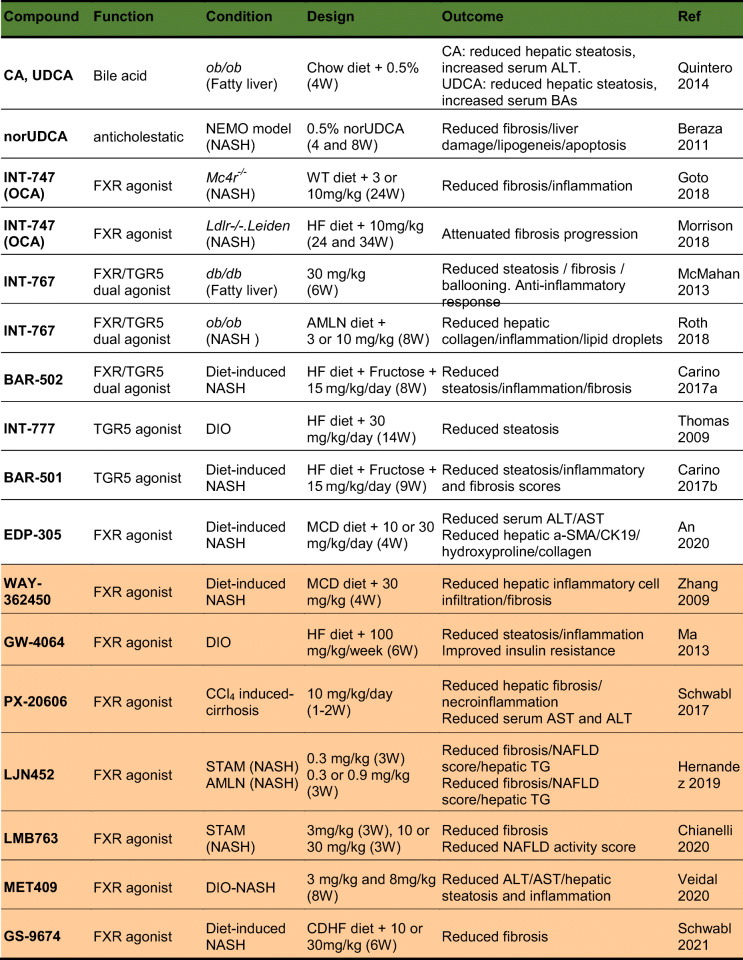
Compounds targeting the bile acid receptors or their enterohepatic circulation and have been used as treatment for MAFLD and its associated comorbidities in pre-clinical studies using animal models. Steroidal (light orange) and non-steroidal (dark orange) compounds

*CA* cholic acid*; UDCA* ursodeoxycholic acid*; BA(s)* bile acid(s)*; ALT* alanine aminotransferase*; AST* aspartate aminotransferase*; norUDCA* nor-ursodeoxycholic acid*; NEMO* NF-kappa-B essential modulator*; OCA* obeticholic acid*; Mc4r* Melanocortin 4 receptor*; WT* Western type*; HF* high fat*; AMLN* Amylin Liver NASH model*; DIO* diet-induced obesity*; MCD* methionine-choline deficient*; a-SMA* a-smooth muscle actin; *CK19* cytokeratin 19; STAM *streptozotocin-administered mice*; *TG* triglycerides; *CDHF* choline-deficient high fat; *LDL* low-density lipoprotein; *HDL* high-density lipoprotein; *GGT* gamma-glutamyltransferase; *T2DM* type 2 diabetes mellitus; *ACC* acetyl-CoA carboxylase; *CCR2/5* chemokine receptor type 2/5; *FGF19* fibroblast growth factor 19; *W* weeks; *M* months

**Table 3:**
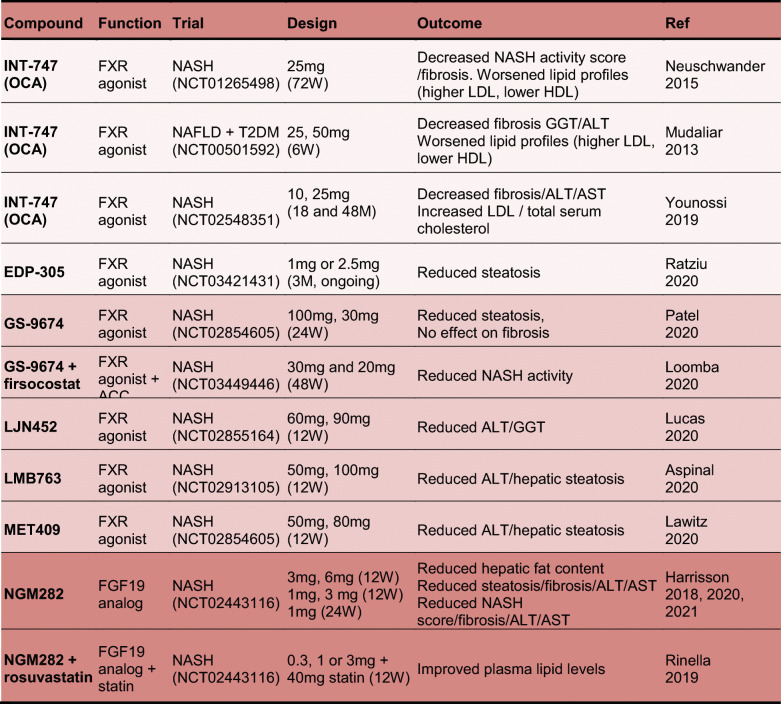
Compounds used alone or in combination with other drugs for clinical trials in patients with metabolic associated steatohepatitis. Steroidal (light purple) and non-steroidal (intermediate purple) agonists of bile acid receptors as well as FGF19 analogs (dark purple)

## Conclusion and future perspectives

In recent years, the importance of bile acids has been demonstrated not only for the regulation of metabolism but also for the function and plasticity of immune cells. Especially in the context of inflammatory bowel diseases, secondary bile acids such as 3-oxo-LCA or isoallo-LCA have been identified, which significantly influence the differentiation of regulatory T cells. The relevance of these newly identified bile acids to inflammatory diseases of the liver has not been investigated to date, and future studies have to investigate whether also these less abundant DCA or LCA derivatives come into contact with parenchymal and immune cells of the liver at a functionally effective concentration.

In this review article, we have not discussed the fundamentally important role of the composition of the gut microbiome in the production of secondary bile acids [103]. However, a recently published study should be mentioned here, since bacteria can apparently also colonize the biliary system under certain conditions, which might be especially important for the etiology of PSC and PBC. In this study, the presence of the gram-positive bacteria *Enterococcus* in the biliary system was associated with the production of potentially harmful bile acids [104]. Future studies will reveal whether secondary bile acids produced locally in the biliary tract might modulate the immune system and/or cholangiocytes, thus critically influencing the progression of inflammatory and metabolic liver diseases. In consequence of their important functions in the regulation of immune cell responses and metabolism, the specific activation of bile acid receptor–dependent signaling pathways has enormous therapeutic potential. Clinical trials of synthetic bile acid derivatives for the treatment of NASH, PBC, and PSC are quite promising [7], although due to the expression of the various bile acid receptors, the occurrence of systemic metabolic and inflammatory side effects should be carefully monitored. Future studies will show whether, for example, the UDCA derivative 24-norUDCA is suitable for the permanent and safe treatment of hitherto untreatable PSC [105]. In this context, the development of cell type–specific or dual receptor agonists could potentially be a way to prevent the development of side effects.

## References

[1] Russell DW (2003). The enzymes, regulation, and genetics of bile acid synthesis. Annu Rev Biochem.

[2] Hofmann AF, Hagey LR (2014). Key discoveries in bile acid chemistry and biology and their clinical applications: history of the last eight decades. J Lipid Res.

[3] Groen AK, Kuipers F (2013). Bile acid look-alike controls life span in C. elegans. Cell Metab.

[4] Lefebvre P, Cariou B, Lien F, Kuipers F, Staels B (2009). Role of bile acids and bile acid receptors in metabolic regulation. Physiol Rev.

[5] Ahmad TR, Haeusler RA (2019). Bile acids in glucose metabolism and insulin signalling - mechanisms and research needs. Nat Rev Endocrinol.

[6] Chiang JYL, Ferrell JM (2019). Bile acids as metabolic regulators and nutrient sensors. Annu Rev Nutr.

[7] Fiorucci S, Distrutti E, Carino A, Zampella A, Biagioli M (2021). Bile acids and their receptors in metabolic disorders. Prog Lipid Res.

[8] Keitel V, Stindt J, Haussinger D (2019). Bile acid-activated receptors: GPBAR1 (TGR5) and other G protein-coupled receptors. Handb Exp Pharmacol.

[9] Shin DJ, Wang L (2019). Bile Acid-Activated Receptors: A Review on FXR and Other Nuclear Receptors. Handb Exp Pharmacol.

[10] Cai X, Young GM, Xie W (2021). The xenobiotic receptors PXR and CAR in liver physiology, an update, Biochimica et biophysica acta. Mol Basis Dis.

[11] Arab JP, Karpen SJ, Dawson PA, Arrese M, Trauner M (2017). Bile acids and nonalcoholic fatty liver disease: Molecular insights and therapeutic perspectives. Hepatology.

[12] Roberts SB, Ismail M, Kanagalingam G, Mason AL, Swain MG, Vincent C, Yoshida EM, Tsien C, Flemming JA, Janssen HLA, Hirschfield GM, Hansen BE, Gulamhusein AF (2020). D. Canadian Network for Autoimmune Liver, Real-world effectiveness of obeticholic acid in patients with primary biliary cholangitis. Hepatol Commun.

[13] Ali AH, Lindor KD (2016). Obeticholic acid for the treatment of primary biliary cholangitis. Expert Opin Pharmacother.

[14] Steiner C, Othman A, Saely CH, Rein P, Drexel H, von Eckardstein A, Rentsch KM (2011). Bile acid metabolites in serum: intraindividual variation and associations with coronary heart disease, metabolic syndrome and diabetes mellitus. PLoS One.

[15] Chen L, van den Munckhof ICL, Schraa K, Ter Horst R, Koehorst M, van Faassen M, van der Ley C, Doestzada M, Zhernakova DV, Kurilshikov A, Bloks VW, Groen AK, Riksen NP, Rutten JHW, Joosten LAB, Wijmenga C, Zhernakova A, Netea MG, Fu J, Kuipers F, P. Human Functional Genomics (2020). Genetic and microbial associations to plasma and fecal bile acids in obesity relate to plasma lipids and liver fat content. Cell Rep.

[16] Haeusler RA, Astiarraga B, Camastra S, Accili D, Ferrannini E (2013). Human insulin resistance is associated with increased plasma levels of 12alpha-hydroxylated bile acids. Diabetes.

[17] de Boer JF, Bloks VW, Verkade E, Heiner-Fokkema MR, Kuipers F (2018). New insights in the multiple roles of bile acids and their signaling pathways in metabolic control. Curr Opin Lipidol.

[18] Ferrell JM, Chiang JYL (2019). Understanding bile acid signaling in diabetes: from pathophysiology to therapeutic targets. Diabetes Metab J.

[19] Takahashi S, Fukami T, Masuo Y, Brocker CN, Xie C, Krausz KW, Wolf CR, Henderson CJ, Gonzalez FJ (2016). Cyp2c70 is responsible for the species difference in bile acid metabolism between mice and humans. J Lipid Res.

[20] de Boer JF, de Vries HD, Palmiotti A, Li R, Doestzada M, Hoogerland JA, Fu J, La Rose AM, Westerterp M, Mulder NL, Hovingh MV, Koehorst M, Kloosterhuis NJ, Wolters JC, Bloks VW, Haas JT, Dombrowicz D, Staels B, van de Sluis B, Kuipers F (2021). Cholangiopathy and biliary fibrosis in Cyp2c70-deficient mice are fully reversed by ursodeoxycholic acid. Cell Mol Gastroenterol Hepatol.

[21] Honda A, Miyazaki T, Iwamoto J, Hirayama T, Morishita Y, Monma T, Ueda H, Mizuno S, Sugiyama F, Takahashi S, Ikegami T (2020). Regulation of bile acid metabolism in mouse models with hydrophobic bile acid composition. J Lipid Res.

[22] Straniero S, Laskar A, Savva C, Hardfeldt J, Angelin B, Rudling M (2020). Of mice and men: murine bile acids explain species differences in the regulation of bile acid and cholesterol metabolism. J Lipid Res.

[23] Schubert K, Olde Damink SWM, von Bergen M, Schaap FG (2017). Interactions between bile salts, gut microbiota, and hepatic innate immunity. Immunol Rev.

[24] Kawamata Y, Fujii R, Hosoya M, Harada M, Yoshida H, Miwa M, Fukusumi S, Habata Y, Itoh T, Shintani Y, Hinuma S, Fujisawa Y, Fujino M (2003). A G protein-coupled receptor responsive to bile acids. J Biol Chem.

[25] Song X, Sun X, Oh SF, Wu M, Zhang Y, Zheng W, Geva-Zatorsky N, Jupp R, Mathis D, Benoist C, Kasper DL (2020). Microbial bile acid metabolites modulate gut RORgamma(+) regulatory T cell homeostasis. Nature.

[26] Hang S, Paik D, Yao L, Kim E, Trinath J, Lu J, Ha S, Nelson BN, Kelly SP, Wu L, Zheng Y, Longman RS, Rastinejad F, Devlin AS, Krout MR, Fischbach MA, Littman DR, Huh JR (2019). Bile acid metabolites control TH17 and Treg cell differentiation. Nature.

[27] Campbell C, McKenney PT, Konstantinovsky D, Isaeva OI, Schizas M, Verter J, Mai C, Jin WB, Guo CJ, Violante S, Ramos RJ, Cross JR, Kadaveru K, Hambor J, Rudensky AY (2020). Bacterial metabolism of bile acids promotes generation of peripheral regulatory T cells. Nature.

[28] Chiang JY (2009). Bile acids: regulation of synthesis. J Lipid Res.

[29] Russell DW (2009). Fifty years of advances in bile acid synthesis and metabolism. J Lipid Res.

[30] Thakare R, Alamoudi JA, Gautam N, Rodrigues AD, Alnouti Y (2018). Species differences in bile acids I. Plasma and urine bile acid composition. J Appl Toxicol.

[31] Perino A, Demagny H, Velazquez-Villegas L, Schoonjans K (2021). Molecular physiology of bile acid signaling in health, disease, and aging. Physiol Rev.

[32] Stellaard F, Sackmann M, Berr F, Paumgartner G (1987). Simultaneous determination of pool sizes and fractional turnover rates, of deoxycholic acid, cholic acid and chenodeoxycholic acid in man by isotope dilution with 2H and 13C labels and serum sampling. Biomed Environ Mass Spectrom.

[33] Koopman BJ, Kuipers F, Bijleveld CM, van der Molen JC, Nagel GT, Vonk RJ, Wolthers BG (1988). Determination of cholic acid and chenodeoxycholic acid pool sizes and fractional turnover rates by means of stable isotope dilution technique, making use of deuterated cholic acid and chenodeoxycholic acid. Clin Chim Acta.

[34] Kuipers F, de Boer JF, Staels B (2020). Microbiome modulation of the host adaptive immunity through bile acid modification. Cell Metab.

[35] Chen ML, Huang X, Wang H, Hegner C, Liu Y, Shang J, Eliason A, Diao H, Park H, Frey B, Wang G, Mosure SA, Solt LA, Kojetin DJ, Rodriguez-Palacios A, Schady DA, Weaver CT, Pipkin ME, Moore DD, Sundrud MS (2021). CAR directs T cell adaptation to bile acids in the small intestine. Nature.

[36] Glaser F, John C, Engel B, Hoh B, Weidemann S, Dieckhoff J, Stein S, Becker N, Casar C, Schuran FA, Wieschendorf B, Preti M, Jessen F, Franke A, Carambia A, Lohse AW, Ittrich H, Herkel J, Heeren J, Schramm C, Schwinge D (2019). Liver infiltrating T cells regulate bile acid metabolism in experimental cholangitis. J Hepatol.

[37] Jansen PL, Strautnieks SS, Jacquemin E, Hadchouel M, Sokal EM, Hooiveld GJ, Koning JH, De Jager-Krikken A, Kuipers F, Stellaard F, Bijleveld CM, Gouw A, Van Goor H, Thompson RJ, Muller M (1999). Hepatocanalicular bile salt export pump deficiency in patients with progressive familial intrahepatic cholestasis. Gastroenterology.

[38] Yokoda RT, Rodriguez EA (2020). Review: Pathogenesis of cholestatic liver diseases. World J Hepatol.

[39] Zou M, Wang A, Wei J, Cai H, Yu Z, Zhang L, Wang X (2021). An insight into the mechanism and molecular basis of dysfunctional immune response involved in cholestasis. Int Immunopharmacol.

[40] Banales JM, Huebert RC, Karlsen T, Strazzabosco M, LaRusso NF, Gores GJ (2019). Cholangiocyte pathobiology, Nature reviews. Gastroenterol Hepatol.

[41] Adams DH, Eksteen B (2006). Aberrant homing of mucosal T cells and extra-intestinal manifestations of inflammatory bowel disease. Nat Rev Immunol.

[42] Gulamhusein AF, Hirschfield GM (2020). Primary biliary cholangitis: pathogenesis and therapeutic opportunities. Nat Rev Gastroenterol Hepatol.

[43] Cameron RG, Blendis LM, Neuman MG (2001). Accumulation of macrophages in primary sclerosing cholangitis. Clin Biochem.

[44] Schmucker DL, Ohta M, Kanai S, Sato Y, Kitani K (1990). Hepatic injury induced by bile salts: correlation between biochemical and morphological events. Hepatology.

[45] Afonso MB, Rodrigues PM, Simao AL, Ofengeim D, Carvalho T, Amaral JD, Gaspar MM, Cortez-Pinto H, Castro RE, Yuan J, Rodrigues CM (2016). Activation of necroptosis in human and experimental cholestasis. Cell Death Dis.

[46] Chen R, Hou W, Zhang Q, Kang R, Fan XG, Tang D (2013). Emerging role of high-mobility group box 1 (HMGB1) in liver diseases. Mol Med.

[47] Calmus Y, Arvieux C, Gane P, Boucher E, Nordlinger B, Rouger P, Poupon R (1992). Cholestasis induces major histocompatibility complex class I expression in hepatocytes. Gastroenterology.

[48] Allen K, Jaeschke H, Copple BL (2011). Bile acids induce inflammatory genes in hepatocytes: a novel mechanism of inflammation during obstructive cholestasis. Am J Pathol.

[49] Gujral JS, Liu J, Farhood A, Jaeschke H (2004). Reduced oncotic necrosis in Fas receptor-deficient C57BL/6J-lpr mice after bile duct ligation. Hepatology.

[50] Polzien F, Ramadori G (1996). Increased intercellular adhesion molecule-1 serum concentration in cholestasis. J Hepatol.

[51] Podevin P, Calmus Y, Bonnefis MT, Veyrunes C, Chereau C, Poupon R (1995). Effect of cholestasis and bile acids on interferon-induced 2',5'-adenylate synthetase and NK cell activities. Gastroenterology.

[52] Tacke F (2017). Targeting hepatic macrophages to treat liver diseases. J Hepatol.

[53] Sturm E, Havinga R, Baller JF, Wolters H, van Rooijen N, Kamps JA, Verkade HJ, Karpen SJ, Kuipers F (2005). Kupffer cell depletion with liposomal clodronate prevents suppression of Ntcp expression in endotoxin-treated rats. J Hepatol.

[54] Harms MH, van Buuren HR, Corpechot C, Thorburn D, Janssen HLA, Lindor KD, Hirschfield GM, Pares A, Floreani A, Mayo MJ, Invernizzi P, Battezzati PM, Nevens F, Ponsioen CY, Mason AL, Kowdley KV, Lammers WJ, Hansen BE, van der Meer AJ (2019). Ursodeoxycholic acid therapy and liver transplant-free survival in patients with primary biliary cholangitis. J Hepatol.

[55] Pares A, Caballeria L, Rodes J (2006). Excellent long-term survival in patients with primary biliary cirrhosis and biochemical response to ursodeoxycholic Acid. Gastroenterology.

[56] Vesterhus M, Karlsen TH (2020). Emerging therapies in primary sclerosing cholangitis: pathophysiological basis and clinical opportunities. J Gastroenterol.

[57] Lindor KD, Kowdley KV, Luketic VA, Harrison ME, McCashland T, Befeler AS, Harnois D, Jorgensen R, Petz J, Keach J, Mooney J, Sargeant C, Braaten J, Bernard T, King D, Miceli E, Schmoll J, Hoskin T, Thapa P, Enders F (2009). High-dose ursodeoxycholic acid for the treatment of primary sclerosing cholangitis. Hepatology.

[58] Beuers U, Spengler U, Zwiebel FM, Pauletzki J, Fischer S, Paumgartner G (1992). Effect of ursodeoxycholic acid on the kinetics of the major hydrophobic bile acids in health and in chronic cholestatic liver disease. Hepatology.

[59] Beuers U, Hohenester S, de Buy Wenniger LJ, Kremer AE, Jansen PL, Elferink RP (2010). The biliary HCO(3)(-) umbrella: a unifying hypothesis on pathogenetic and therapeutic aspects of fibrosing cholangiopathies. Hepatology.

[60] Gurantz D, Schteingart CD, Hagey LR, Steinbach JH, Grotmol T, Hofmann AF (1991). Hypercholeresis induced by unconjugated bile acid infusion correlates with recovery in bile of unconjugated bile acids. Hepatology.

[61] Ozcan U, Yilmaz E, Ozcan L, Furuhashi M, Vaillancourt E, Smith RO, Gorgun CZ, Hotamisligil GS (2006). Chemical chaperones reduce ER stress and restore glucose homeostasis in a mouse model of type 2 diabetes. Science.

[62] Terasaki S, Nakanuma Y, Ogino H, Unoura M, Kobayashi K (1991). Hepatocellular and biliary expression of HLA antigens in primary biliary cirrhosis before and after ursodeoxycholic acid therapy. Am J Gastroenterol.

[63] Poupon R (2012). Ursodeoxycholic acid and bile-acid mimetics as therapeutic agents for cholestatic liver diseases: an overview of their mechanisms of action. Clin Res Hepatol Gastroenterol.

[64] Nishigaki Y, Ohnishi H, Moriwaki H, Muto Y (1996). Ursodeoxycholic acid corrects defective natural killer activity by inhibiting prostaglandin E2 production in primary biliary cirrhosis. Dig Dis Sci.

[65] Verbeke L, Farre R, Verbinnen B, Covens K, Vanuytsel T, Verhaegen J, Komuta M, Roskams T, Chatterjee S, Annaert P, Vander Elst I, Windmolders P, Trebicka J, Nevens F, Laleman W (2015). The FXR agonist obeticholic acid prevents gut barrier dysfunction and bacterial translocation in cholestatic rats. Am J Pathol.

[66] Hirschfield GM, Mason A, Luketic V, Lindor K, Gordon SC, Mayo M, Kowdley KV, Vincent C, Bodhenheimer HC, Pares A, Trauner M, Marschall HU, Adorini L, Sciacca C, Beecher-Jones T, Castelloe E, Bohm O, Shapiro D (2015). Efficacy of obeticholic acid in patients with primary biliary cirrhosis and inadequate response to ursodeoxycholic acid. Gastroenterology.

[67] Kowdley KV, Luketic V, Chapman R, Hirschfield GM, Poupon R, Schramm C, Vincent C, Rust C, Pares A, Mason A, Marschall HU, Shapiro D, Adorini L, Sciacca C, Beecher-Jones T, Bohm O, Pencek R, Jones D, P.B.C.M.S.G. Obeticholic Acid (2018, 1890-1902) A randomized trial of obeticholic acid monotherapy in patients with primary biliary cholangitis. Hepatology 67(5) 10.1002/hep.29569 PMC5947631 29023915

[68] Eslam M, Sanyal AJ, George J, P. International Consensus (2020). MAFLD: A Consensus-Driven Proposed Nomenclature for Metabolic Associated Fatty Liver Disease. Gastroenterology.

[69] Loomba R, Sanyal AJ (2013). The global NAFLD epidemic. Nat Rev Gastroenterol Hepatol.

[70] Cohen JC, Horton JD, Hobbs HH (2011). Human fatty liver disease: old questions and new insights. Science.

[71] Bechmann LP, Hannivoort RA, Gerken G, Hotamisligil GS, Trauner M, Canbay A (2012). The interaction of hepatic lipid and glucose metabolism in liver diseases. J Hepatol.

[72] Molinaro A, Wahlstrom A, Marschall HU (2018). Role of Bile Acids in Metabolic Control. Trends Endocrinol Metab.

[73] Shapiro H, Kolodziejczyk AA, Halstuch D, Elinav E (2018). Bile acids in glucose metabolism in health and disease. J Exp Med.

[74] Staels B, Fonseca VA (2009). Bile acids and metabolic regulation: mechanisms and clinical responses to bile acid sequestration. Diabetes Care.

[75] Tanaka N, Matsubara T, Krausz KW, Patterson AD, Gonzalez FJ (2012). Disruption of phospholipid and bile acid homeostasis in mice with nonalcoholic steatohepatitis. Hepatology.

[76] Suga T, Yamaguchi H, Ogura J, Shoji S, Maekawa M, Mano N (2019). Altered bile acid composition and disposition in a mouse model of non-alcoholic steatohepatitis. Toxicol Appl Pharmacol.

[77] Ferslew BC, Xie G, Johnston CK, Su M, Stewart PW, Jia W, Brouwer KL, Barritt AST (2015). Altered Bile Acid Metabolome in Patients with Nonalcoholic Steatohepatitis. Dig Dis Sci.

[78] Bechmann LP, Kocabayoglu P, Sowa JP, Sydor S, Best J, Schlattjan M, Beilfuss A, Schmitt J, Hannivoort RA, Kilicarslan A, Rust C, Berr F, Tschopp O, Gerken G, Friedman SL, Geier A, Canbay A (2013). Free fatty acids repress small heterodimer partner (SHP) activation and adiponectin counteracts bile acid-induced liver injury in superobese patients with nonalcoholic steatohepatitis. Hepatology.

[79] Dasarathy S, Yang Y, McCullough AJ, Marczewski S, Bennett C, Kalhan SC (2011). Elevated hepatic fatty acid oxidation, high plasma fibroblast growth factor 21, and fasting bile acids in nonalcoholic steatohepatitis. Eur J Gastroenterol Hepatol.

[80] Kalhan SC, Guo L, Edmison J, Dasarathy S, McCullough AJ, Hanson RW, Milburn M (2011). Plasma metabolomic profile in nonalcoholic fatty liver disease. Metab Clin Exp.

[81] Puri P, Daita K, Joyce A, Mirshahi F, Santhekadur PK, Cazanave S, Luketic VA, Siddiqui MS, Boyett S, Min HK, Kumar DP, Kohli R, Zhou H, Hylemon PB, Contos MJ, Idowu M, Sanyal AJ (2018). The presence and severity of nonalcoholic steatohepatitis is associated with specific changes in circulating bile acids. Hepatology.

[82] Nimer N, Choucair I, Wang Z, Nemet I, Li L, Gukasyan J, Weeks TL, Alkhouri N, Zein N, Tang WHW, Fischbach MA, Brown JM, Allayee H, Dasarathy S, Gogonea V, Hazen SL (2021). Bile acids profile, histopathological indices and genetic variants for non-alcoholic fatty liver disease progression. Metab Clin Exp.

[83] Aranha MM, Cortez-Pinto H, Costa A, da Silva IB, Camilo ME, de Moura MC, Rodrigues CM (2008). Bile acid levels are increased in the liver of patients with steatohepatitis. Eur J Gastroenterol Hepatol.

[84] Lake AD, Novak P, Shipkova P, Aranibar N, Robertson D, Reily MD, Lu Z, Lehman-McKeeman LD, Cherrington NJ (2013). Decreased hepatotoxic bile acid composition and altered synthesis in progressive human nonalcoholic fatty liver disease. Toxicol Appl Pharmacol.

[85] Mouzaki M, Wang AY, Bandsma R, Comelli EM, Arendt BM, Zhang L, Fung S, Fischer SE, McGilvray IG, Allard JP (2016). Bile Acids and Dysbiosis in Non-Alcoholic Fatty Liver Disease. PLoS One.

[86] Legry V, Francque S, Haas JT, Verrijken A, Caron S, Chavez-Talavera O, Vallez E, Vonghia L, Dirinck E, Verhaegen A, Kouach M, Lestavel S, Lefebvre P, Van Gaal L, Tailleux A, Paumelle R, Staels B (2017). Bile Acid Alterations Are Associated With Insulin Resistance, but Not With NASH, in Obese Subjects. J Clin Endocrinol Metab.

[87] Lee G, You HJ, Bajaj JS, Joo SK, Yu J, Park S, Kang H, Park JH, Kim JH, Lee DH, Lee S, Kim W, Ko G (2020). Distinct signatures of gut microbiome and metabolites associated with significant fibrosis in non-obese NAFLD. Nat Commun.

[88] Heeren J, Scheja L (2018). Brown adipose tissue and lipid metabolism. Curr Opin Lipidol.

[89] Raselli T, Hearn T, Wyss A, Atrott K, Peter A, Frey-Wagner I, Spalinger MR, Maggio EM, Sailer AW, Schmitt J, Schreiner P, Moncsek A, Mertens J, Scharl M, Griffiths WJ, Bueter M, Geier A, Rogler G, Wang Y, Misselwitz B (2019). Elevated oxysterol levels in human and mouse livers reflect nonalcoholic steatohepatitis. J Lipid Res.

[90] Worthmann A, John C, Ruhlemann MC, Baguhl M, Heinsen FA, Schaltenberg N, Heine M, Schlein C, Evangelakos I, Mineo C, Fischer M, Dandri M, Kremoser C, Scheja L, Franke A, Shaul PW, Heeren J (2017). Cold-induced conversion of cholesterol to bile acids in mice shapes the gut microbiome and promotes adaptive thermogenesis. Nat Med.

[91] Jiao N, Baker SS, Chapa-Rodriguez A, Liu W, Nugent CA, Tsompana M, Mastrandrea L, Buck MJ, Baker RD, Genco RJ, Zhu R, Zhu L (2018). Suppressed hepatic bile acid signalling despite elevated production of primary and secondary bile acids in NAFLD. Gut.

[92] Govaere O, Cockell S, Tiniakos D, Queen R, Younes R, Vacca M, Alexander L, Ravaioli F, Palmer J, Petta S, Boursier J, Rosso C, Johnson K, Wonders K, Day CP, Ekstedt M, Oresic M, Darlay R, Cordell HJ, Marra F, Vidal-Puig A, Bedossa P, Schattenberg JM, Clement K, Allison M, Bugianesi E, Ratziu V, Daly AK, Anstee QM (2020) Transcriptomic profiling across the nonalcoholic fatty liver disease spectrum reveals gene signatures for steatohepatitis and fibrosis. Sci Transl Med 12(572) 10.1126/scitranslmed.aba4448 33268509

[93] Liu H, Pathak P, Boehme S, Chiang JY (2016). Cholesterol 7alpha-hydroxylase protects the liver from inflammation and fibrosis by maintaining cholesterol homeostasis. J Lipid Res.

[94] Bjursell M, Wedin M, Admyre T, Hermansson M, Bottcher G, Goransson M, Linden D, Bamberg K, Oscarsson J, Bohlooly YM (2013). Ageing Fxr deficient mice develop increased energy expenditure, improved glucose control and liver damage resembling NASH. PLoS One.

[95] Ferrell JM, Pathak P, Boehme S, Gilliland T, Chiang JYL (2019). Deficiency of both farnesoid X receptor and Takeda G protein-coupled receptor 5 exacerbated liver fibrosis in mice. Hepatology.

[96] Schmitt J, Kong B, Stieger B, Tschopp O, Schultze SM, Rau M, Weber A, Mullhaupt B, Guo GL, Geier A (2015). Protective effects of farnesoid X receptor (FXR) on hepatic lipid accumulation are mediated by hepatic FXR and independent of intestinal FGF15 signal. Liver Int.

[97] Wang YD, Chen WD, Yu D, Forman BM, Huang W (2011). The G-protein-coupled bile acid receptor, Gpbar1 (TGR5), negatively regulates hepatic inflammatory response through antagonizing nuclear factor kappa light-chain enhancer of activated B cells (NF-kappaB) in mice. Hepatology.

[98] Schaap FG, Trauner M, Jansen PL (2014). Bile acid receptors as targets for drug development, Nature reviews. Gastroenterol Hepatol.

[99] Thomas C, Pellicciari R, Pruzanski M, Auwerx J, Schoonjans K (2008). Targeting bile-acid signalling for metabolic diseases. Nat Rev Drug Discov.

[100] Neuschwander-Tetri BA, Loomba R, Sanyal AJ, Lavine JE, Van Natta ML, Abdelmalek MF, Chalasani N, Dasarathy S, Diehl AM, Hameed B, Kowdley KV, McCullough A, Terrault N, Clark JM, Tonascia J, Brunt EM, Kleiner DE, Doo E, Network NCR (2015). Farnesoid X nuclear receptor ligand obeticholic acid for non-cirrhotic, non-alcoholic steatohepatitis (FLINT): a multicentre, randomised, placebo-controlled trial. Lancet.

[101] Mudaliar S, Henry RR, Sanyal AJ, Morrow L, Marschall HU, Kipnes M, Adorini L, Sciacca CI, Clopton P, Castelloe E, Dillon P, Pruzanski M, Shapiro D (2013). Efficacy and safety of the farnesoid X receptor agonist obeticholic acid in patients with type 2 diabetes and nonalcoholic fatty liver disease. Gastroenterology.

[102] Younossi ZM, Ratziu V, Loomba R, Rinella M, Anstee QM, Goodman Z, Bedossa P, Geier A, Beckebaum S, Newsome PN, Sheridan D, Sheikh MY, Trotter J, Knapple W, Lawitz E, Abdelmalek MF, Kowdley KV, Montano-Loza AJ, Boursier J, Mathurin P, Bugianesi E, Mazzella G, Olveira A, Cortez-Pinto H, Graupera I, Orr D, Gluud LL, Dufour JF, Shapiro D, Campagna J, Zaru L, MacConell L, Shringarpure R, Harrison S, Sanyal AJ, R.S. Investigators (2019). Obeticholic acid for the treatment of non-alcoholic steatohepatitis: interim analysis from a multicentre, randomised, placebo-controlled phase 3 trial. Lancet.

[103] Li R, Andreu-Sanchez S, Kuipers F, Fu J (2021) Gut microbiome and bile acids in obesity-related diseases, Best practice & research. Clin Endocrinol Metab 101493 10.1016/j.beem.2021.101493 33707081

[104] Liwinski T, Zenouzi R, John C, Ehlken H, Ruhlemann MC, Bang C, Groth S, Lieb W, Kantowski M, Andersen N, Schachschal G, Karlsen TH, Hov JR, Rosch T, Lohse AW, Heeren J, Franke A, Schramm C (2020). Alterations of the bile microbiome in primary sclerosing cholangitis. Gut.

[105] Fickert P, Hirschfield GM, Denk G, Marschall HU, Altorjay I, Farkkila M, Schramm C, Spengler U, Chapman R, Bergquist A, Schrumpf E, Nevens F, Trivedi P, Reiter FP, Tornai I, Halilbasic E, Greinwald R, Prols M, Manns MP, Trauner M, P.S.C.n.S.G. European (2017). norUrsodeoxycholic acid improves cholestasis in primary sclerosing cholangitis. J Hepatol.

